# Role of polyvinylpyrrolidone in the electrochemical performance of Li_2_MnO_3_ cathode for lithium-ion batteries†

**DOI:** 10.1039/c8ra10569c

**Published:** 2019-04-02

**Authors:** Ji-Eun Lee, Min-Cheol Kim, Sang-Hyun Moon, Eun-Soo Kim, Yeon-Kyung Shin, Sojeong Choi, Suk-Hui Kwon, Si-Jin Kim, Hye-Jin Kwon, Kyung-Won Park

**Affiliations:** Department of Chemical Engineering, Soongsil University Seoul 06987 Republic of Korea kwpark@ssu.ac.kr +82-2-812-5378 +82-2-820-0613

## Abstract

While Li_2_MnO_3_ as an over-lithiated layered oxide (OLO) shows a significantly high reversible capacity of 250 mA h g^−1^ in lithium-ion batteries (LIBs), it has critical issues of poor cycling performance and deteriorated high rate performance. In this study, modified OLO cathode materials for improved LIB performance were obtained by heating the as-prepared OLO at different temperatures (400, 500, and 600 °C) in the presence of polyvinylpyrrolidone (PVP) under an N_2_ atmosphere. Compared to the as-prepared OLO, the OLO sample heated at 500 °C with PVP exhibited a high initial discharge capacity of 206 mA h g^−1^ and high rate capability of 111 mA h g^−1^ at 100 mA g^−1^. The superior performance of the OLO sample heated at 500 °C with PVP is attributed to an improved electronic conductivity and Li^+^ ionic motion, resulting from the formation of the graphitic carbon structure and increased Mn^3+^ ratio during the decomposition of PVP.

## Abstract 2

The modified OLO cathode materials for improved LIB performance were obtained by heating the as-prepared OLO in the presence of polyvinylpyrrolidone (PVP) under an N_2_ atmosphere.

## Graphical abstract

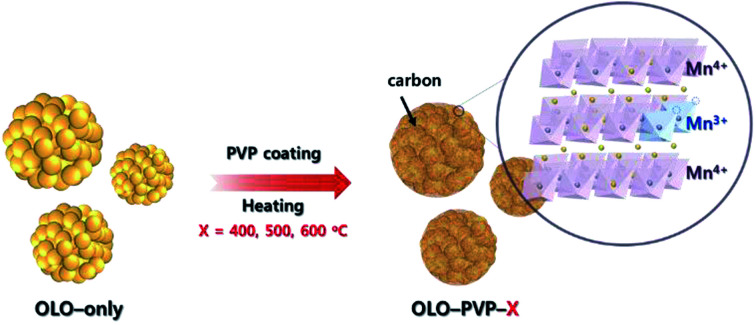

## 1. Introduction

Lithium-ion batteries (LIBs) have been attractive as power sources for portable devices, electric vehicles, and energy storage systems due to their high working voltage, high energy density, and long cycle life.^1–4^ In particular, an important factor affecting the performance in LIBs can be the selection of a cathode material at a low cost, high capacity, and improved stability. The representative cathode candidates are transition metal oxides with relatively low energy densities such as LiCoO_2_ (150 mA h g^−1^), spinel LiMn_2_O_4_ (130 mA h g^−1^), and LiFePO_4_ (160 mA h g^−1^) with a layered, spinel, and olivine structure, respectively.^5–8^ Among these cathode materials, Mn-based structures can be promising due to their abundance, low cost, eco-friendliness, and various valences for high capacity.

In particular, Li_2_MnO_3_ as a Li-rich cathode material or over-lithiated layered oxide (OLO), exhibits a theoretical capacity of 458 mA h g^−1^ and a significantly high reversible capacity of 250 mA h g^−1^.^9,10^ Li_2_MnO_3_ has a Mn^4+^ layered structure consisting of Mn^4+^ layers, expressing as Li[Li_1/3_Mn_2/3_]O_2_. In the octahedral sites within the layered structure, Li^+^ and Mn^4+^ ions can be occupied with a relative ratio of 1 : 2.^11,12^ Moreover, Li^+^ ions in Li_2_MnO_3_ as a cathode material can be extracted at a high potential of >4.5 V *vs.* Li/Li^+^.^13,14^ The charge compensation during the 1st charge process can specifically occur due to the simultaneous loss of oxygen with the extraction of Li^+^ ions.^15–17^ However, the poor cycle stability of Li_2_MnO_3_ can result from phase transformation in electrodes during cycling and side reaction between electrode and electrolyte at >4.5 V.^18–20^ Furthermore, a significantly low electrical and ionic conductivity of Li_2_MnO_3_ can lead to a deteriorated rate capability.^21^ Thus, to enhance the performance of the LIBs with Li_2_MnO_3_ as a cathode, substitutions of Mn with 2nd elements such as Fe or Ru or surface coating with stable oxides such as TiO_2_ and Al_2_O_3_ have been performed.^22–25^ In particular, the surface modification of Li_2_MnO_3_ as an active material with carbon materials can result in an improved electrical conductivity and suppression of the side reaction between the electrode and electrolyte.^26–29^ Furthermore, a post-carbon coating process can be more effective than an *in situ* carbon coating process, in which the as-formed carbon can be combusted during the air heating process. The strategy to improve the electrochemical performance of Li_2_MnO_3_ in LIBs is to control the ratio between Mn^4+^ and Mn^3+^ under a reducing atmosphere.

**Fig. 1:**
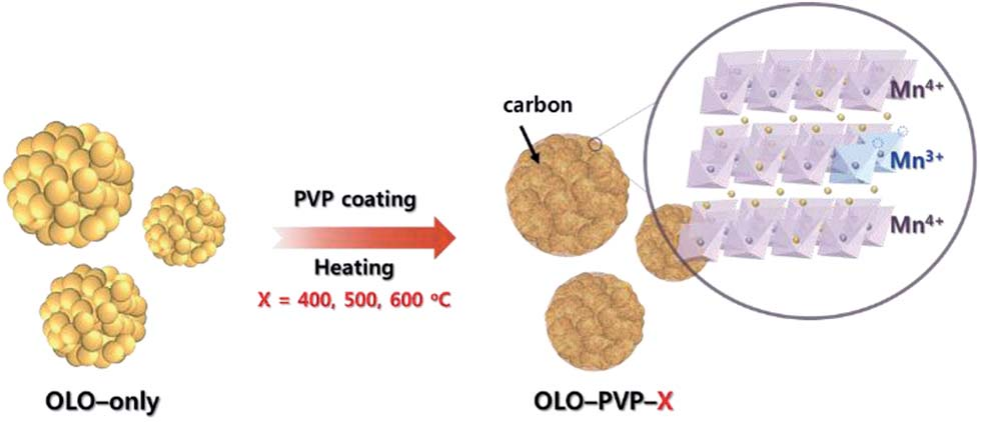
Schematic illustration of synthesis of the samples (OLO-PVP-*X*) heated with Li_2_MnO_3_ under an N_2_ atmosphere at 400, 500, and 600 °C.

In this study, polyvinylpyrrolidone (PVP, (C_6_H_9_NO)_*n*_) was utilized as an additive for a reducing agent and carbon source in a post surface process of Li_2_MnO_3_ as a cathode material to obtain improved stability and rate capability in the LIBs. During the post surface process with PVP, the decomposition of PVP can cause carbon material to form on the electrode surface, resulting in an improved electrical conductivity of the active material (Fig. 1).^30–32^ Moreover, a reducing atmosphere, generated by the decomposition of PVP, can induce an oxygen loss in the electrode surface and produce Mn^3+^ slightly reduced with Mn^4+^ to maintain the charge neutrality (Fig. 1). Furthermore, the distortion of Mn–O_6_ octahedron structure can occur due to the ionic radius difference between Mn^3+^(0.66 Å) and Mn^4+^(0.53 Å), resulting in decreased resistance of Li^+^ ion transport.^33–35^ Despite the drawback of the Li_2_MnO_3_ cathode such as the Jahn–Teller effect, an appropriate portion of Mn^3+^ in the Mn-based cathode materials can lead to an improved transport of Li ion and decreased surface resistance, representing an enhanced electrochemical performance.^36–38^

## 2. Experimental

### 2.1 Synthesis of Li_2_MnO_3_

Pluronic acid (10 g, P123, (PEO)_20_–(PPO)_70_–(PEO)_20_, Aldrich) as a polymer template was mixed in cyclohexane (80 g, Aldrich) and *n*-butanol (9.6 g, BuOH, Aldrich) as a co-surfactant with lithium dodecylsulfate (0.45 g, LDS, Aldrich) for 4 h. Then, 10 mL of 2.8 M LiNO_3_ and 10 mL of 1.0 M Mn(NO_3_)_2_ were mixed in the solution with 0.2 g Ketjen black while continuously stirring for 20 h. The gel state sample was obtained by heating the mixed solution at 130 °C for 4 h. The sample was heated in an air atmosphere at 300 °C for 6 h and then at 600 °C for 10 h. The resulting powder was washed in acetone with continuous stirring for 12 h in order to remove impurities. Li_2_MnO_3_ as a final product was obtained in a 60 °C vacuum oven for 24 h (denoted as OLO-only) (Fig. 1).^39,40^

### 2.2 Synthesis of Li_2_MnO_3_–P5VP

0.5 g Li_2_MnO_3_ was ultrasonicated in 20 mL de-ionized (DI) water for 1 h and 0.125 g polyvinylpyrrolidone (PVP, MW = 29 000, Aldrich) was then added with 30 mL DI water. The mixed solution was heated in an 80 °C water bath for 2 h. The solution was then filtered and dried in a 60 °C vacuum oven for 24 h. The dried sample was heated in an air atmosphere at 200 °C for 2 h in order to remove impurities and in an N_2_ atmosphere at 400–600 °C for 5 h. The samples heated at 400, 500, and 600 °C were denoted as OLO-PVP-400, OLO-PVP-500, and OLO-PVP-600, respectively (Fig. 1).^41,42^

### 2.3 Materials characterization

To characterize the crystal structure of the samples, an X-ray diffractometer (XRD, Bruker, D2 PHASER) with a Cu K_α_ source (*λ* = 0.15418 nm) and an Ni filter was used under an operating voltage of 30 kV and a working current of 10 mA. The morphology and structure of the samples were analyzed using a scanning electron microscope (SEM, ZEISS, GeminiSEM 300, 1.50 kV) and transmission electron microscope (TEM, JEOL, JEM-ARM 200F, 200 kV). The composition of the sample was confirmed using energy-dispersive X-ray spectroscopy (EDX, JEM-ARM 200F). The powder was ultrasonically dispersed in DI water and the sample for TEM analysis was prepared by dropping 10 μL suspension solution on a Ni grid. Thermogravimetric analysis (TGA, TA Instrument, SDT Q600) of the samples was performed at a temperature range of 30–800 °C in an air atmosphere. The specific surface area and pore size distribution of the samples were measured using an N_2_ adsorption analyzer (Micromeritics, ASAP 2020). To confirm the chemical compositions and states of the samples, X-ray photoelectron spectroscopy (XPS, Thermo VG, UK) was performed with a beam source of Al K_α_ (1486.6 eV) and a power of 200 W under a chamber pressure of 4.8 × 10^−9^ torr.

### 2.4 Electrochemical characterization

The electrochemical performances of the samples as cathodes in LIBs were evaluated using coin-type cells (size 2032, Hohsen Corporation). The slurry for the cathode was prepared by mixing 80 wt% cathode powder with 10 wt% polyvinylidene difluoride (PVDF) and 10 wt% Ketjen black in 1-methyl-2 pyrrolidinone (NMP) solvent. The slurry was coated on an aluminum foil current collector using a doctor blade method and dried in a 110 °C oven for 12 h. The dried electrode was cut down to a diameter of 1.3 cm. The cell assembly was performed in an Ar-filled glove box (<5 ppm, H_2_O and O_2_) using the resulting electrode as a working electrode and lithium metal (FMC Corporation) as a counter electrode. Polyethylene (Wellcos) and 1.1 M LiPF_6_ in ethylene carbonate : diethyl carbonate (1 : 1) (Techno Semichem) were used as a porous separator and electrolyte, respectively. The assembled cell was evaluated at 25 °C using a multichannel battery tester (WBCS300L, Wonatech Co.). The charge/discharge test was performed at a current density of 20 mA g^−1^ in the potential range of 2.0–4.8 V *vs.* Li/Li^+^ for 50 cycles. To compare the high-rate performances, the charge/discharging test was carried out at different current densities of 20–100 mA g^−1^ for 5 cycles. Cyclic voltammograms (CVs) were obtained in the potential range of 2.0–4.8 V with a scan rate of 0.1 mV s^−1^. Galvanostatic intermittent titration technique (GITT) analysis was carried out in the potential range of 2.0–4.8 V *vs.* Li/Li^+^ at a current density of 10 mA g^−1^ with a 10 min-pulse and a 10 min-relaxation.

## 3. Results and discussion

**Fig. 2:**
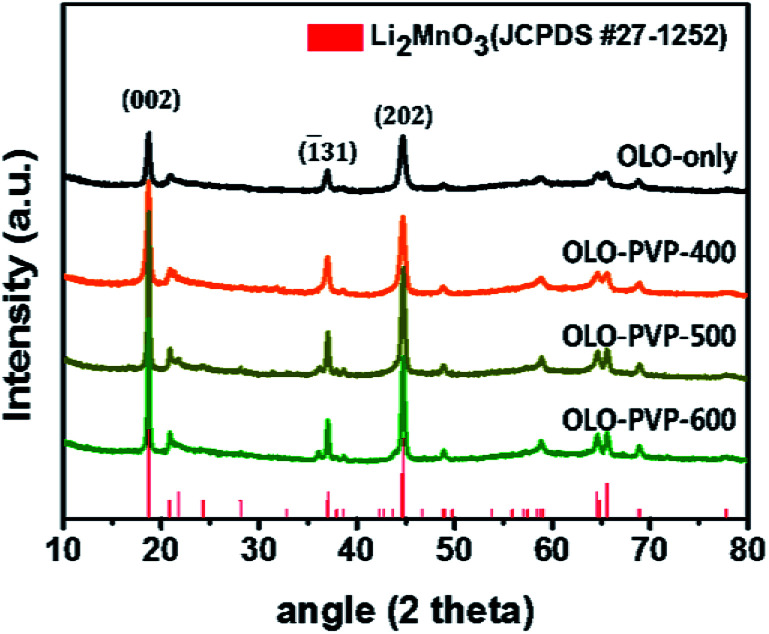
XRD patterns of the as-prepared samples.

Fig. 2 shows the XRD patterns of the as-prepared OLO-only, OLO-PVP-400, OLO-PVP-500, and OLO-PVP-600 samples. All the samples exhibit typical XRD patterns corresponding to the crystal structure of Li_2_MnO_3_ (JCPDS #27-1252) with *C*2/*m* space group without other impurities or structures. In particular, the XRD peaks at 18.71°, 21.77°, 37.06°, 44.6°, 44.81°, 64.53°, and 65.60° correspond to the (002), (1̄11), (1̄31), (202), (1̄33), (135), and (060) planes, respectively. The crystal structure of OLO-PVP-400, OLO-PVP-500, and OLO-PVP-600 prepared with PVP was identical to that of OLO-only. This demonstrates that the original crystal structure of Li_2_MnO_3_ can be maintained during the heating process in the presence of PVP. Furthermore, with increasing reaction temperature from 400 to 600 °C, the XRD peaks in the samples became sharper, representing the increased particle size of the samples. On the other hand, no XRD pattern associated with the carbon phase was detected due to the relatively slight amount of carbon in the samples.^43–45^

**Fig. 3:**
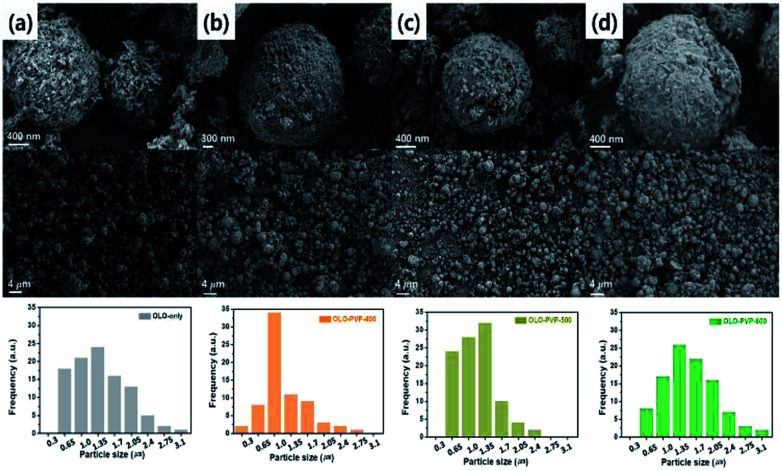
SEM images and size distributions (insets) of (a) OLO-only, (b) OLO-PVP-400, (c) OLO-PVP-500, and (d) OLO-PVP-600.

**Fig. 4:**
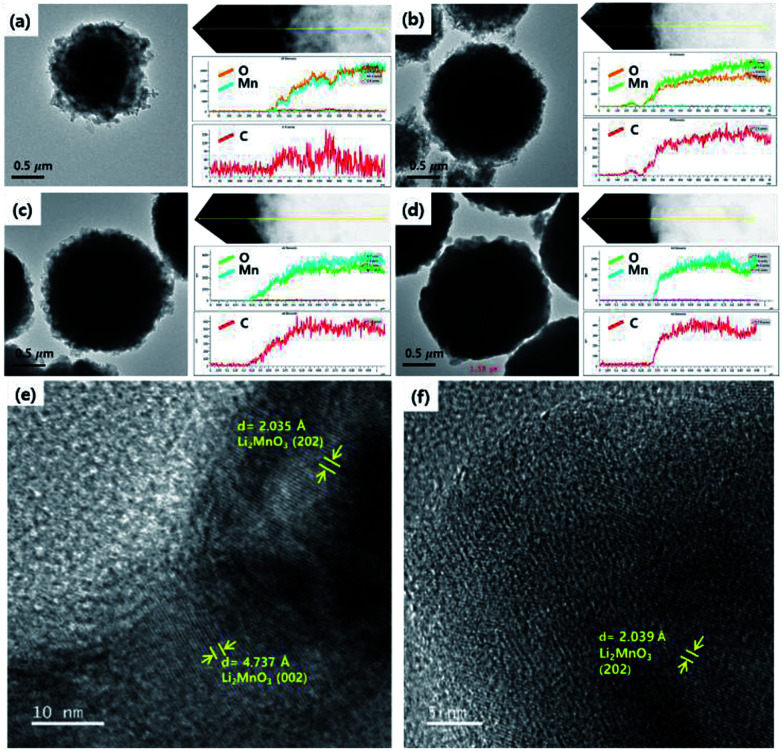
TEM images and line profiles of (a) OLO-only, (b) OLO-PVP-400, (c) OLO-PVP-500, and (d) OLO-PVP-600. High-resolution TEM mages of (e) OLO-PVP-500 and (f) OLO-PVP-600.

The size and morphology of the as-prepared samples were characterized using SEM analysis (Fig. 3). The average particle sizes of OLO-only, OLO-PVP-400, OLO-PVP-500, and OLO-PVP-600 were 1.3, 1.0, 1.4, and 1.7 μm, respectively, as indicated in the size histograms. Compared to OLO-only with a relatively coarse surface, the particle size of OLO-PVP-400 decreased, which might be attributed to the shrinkage of the particle resulting from the reduction of the inner pores during the heating process. However, as the heating temperature increased from 400 to 600 °C, the size of the samples increased with a smooth surface. This demonstrates that the overall morphology of the samples heated in the presence of PVP can be maintained with a transition from coarse to smooth surface. Furthermore, as shown in the TEM images (Fig. 4(a)–(d)), the samples were spherical, which is in agreement with the SEM images. Moreover, the homogeneous surface distribution of C, O and Mn in OLO-PVP-400, OLO-PVP-500, and OLO-PVP-600 could be observed using line profile analysis. In particular, the *d*-spacings of the planes corresponding to the layered Li_2_MnO_3_ structure were identified (Fig. 4(e) and (f)).

**Fig. 5:**
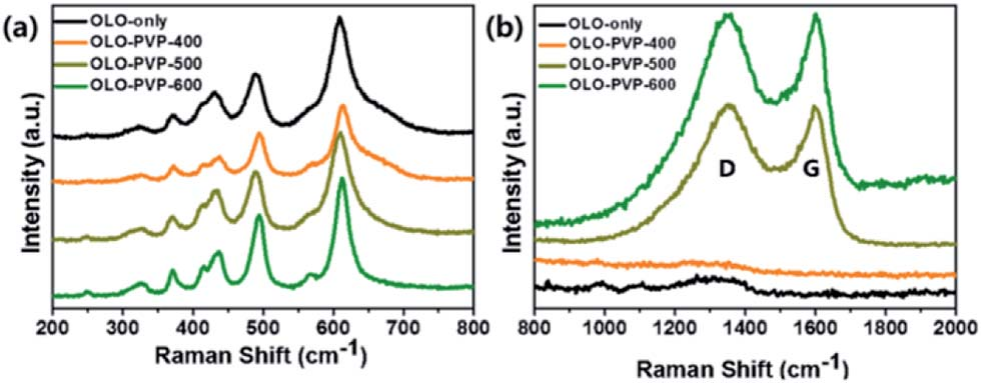
Raman spectra of the samples in the Raman shifts of (a) 200–800 cm^−1^ and (b) 800–2000 cm^−1^.

To confirm the crystal structure of the samples prepared in the presence of PVP, Raman analysis was performed, with the results shown in Fig. 5. The peaks at 615, 560, 496, 438, 416, 372, 325, and 250 cm^−1^ in the Raman shift between 200 and 800 cm^−1^ correspond to the Li–O and Mn–O bondings (Fig. 5(a)), which is in accordance with a monoclinic Li_2_MnO_3_ structure.^12,13,38,44^ In addition, the Raman peaks at ∼1350 and ∼1594 cm^−1^ corresponding to the D- and G-bands, respectively, imply the disordered and graphitic carbon structure, respectively. As shown in Fig. 5(b), compared to the OLO-PVP-400, OLO-PVP-500, and OLO-PVP-600 had two characteristic peaks associated with the D- and G-bands, which are related to the scattering disordered and ordered sp^2^ bonding carbon structures, respectively, demonstrating the complete decomposition of PVP to the carbon phases at >500 °C. The relative intensity ratios of *I*_D_ to *I*_G_, for OLO-PVP-500 and OLO-PVP-600 were 1.01 and 0.98, respectively, demonstrating the enhanced electrical conductivity of the OLO samples heated at >500 °C with PVP.^46–49^

**Fig. 6:**
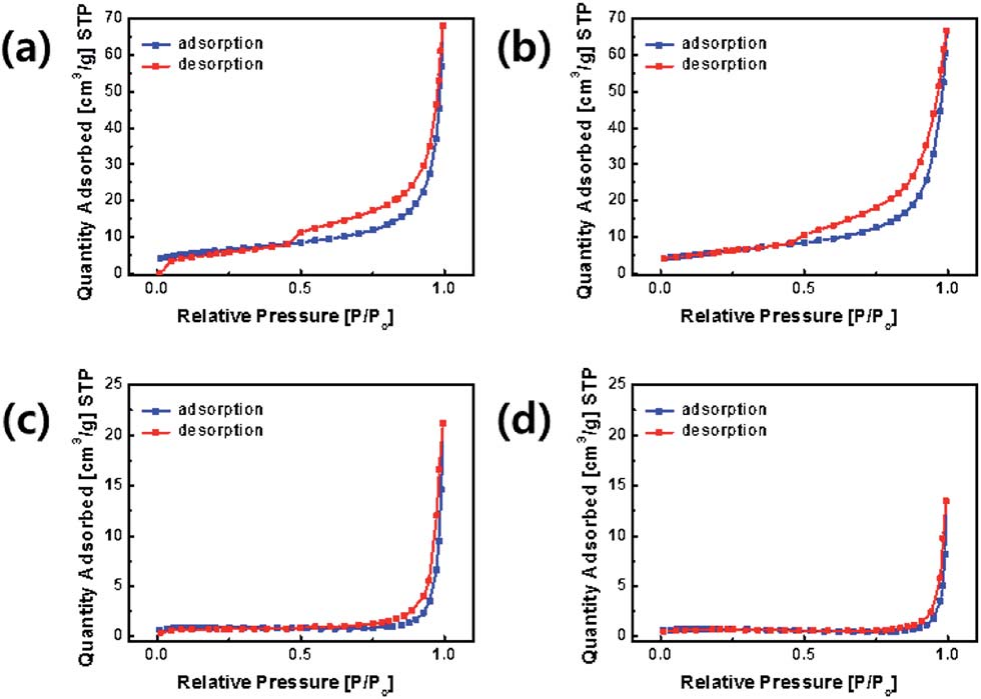
Nitrogen adsorption–desorption curves of (a) OLO-only, (b) OLO-PVP-400, (c) OLO-PVP-500, and (d) OLO-PVP-600.

The nitrogen adsorption–desorption curves of the samples are shown in Fig. 6. The specific surface areas of OLO-only, OLO-PVP-400, OLO-PVP-500, and OLO-PVP-600 were 21.7 20.9, 3.2, and 2.5 m^2^ g^−1^, respectively. OLO-only and OLO-PVP-400 exhibited a type IV curve with average pore sizes of 14.6 and 13.9 nm, respectively, maintaining a mesoporous structure formed due to Ketjen black particles in the synthetic process (Fig. S1†).^40^ On the other hand, OLO-PVP-500 and OLO-PVP-600 exhibited non-porous characteristics with a relatively low surface area due to the complete decomposition of PVP at 500–600 °C. However, the relatively low surface area of OLO-PVP-500 and OLO-PVP-600 can have a stable electrochemical LIB performance, suppressing the decomposition of the active materials at a high voltage during the charge/discharge process.^26–28^

**Fig. 7:**
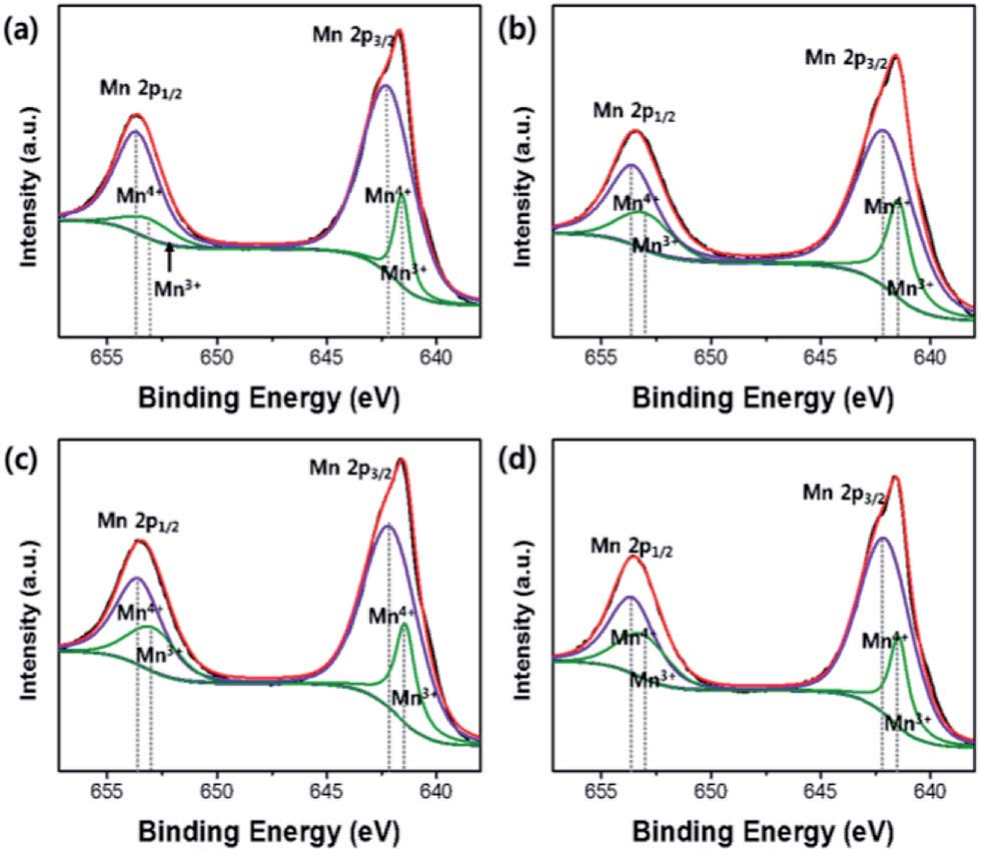
Mn2p XPS spectra of (a) OLO-only, (b) OLO-PVP-400, (c) OLO-PVP-500, and (d) OLO-PVP-600.

The carbon contents in OLO-only, OLO-PVP-400, OLO-PVP-500, and OLO-PVP-600 were 36.4, 32.2, 43.7, and 38.4 at%, respectively (Table 1). The presence of carbon in OLO-only might result from the decomposition of polymers for micelle structure. The oxygen contents in OLO-only, OLO-PVP-400, OLO-PVP-500, and OLO-PVP-600 were 35.4, 40.9, 32.9, and 34.8 at%, respectively. When compared to OLO-only, the oxygen content in OLO-PVP-400 increased due to the heating process in the presence of PVP ((C_6_H_9_NO)_*n*_). However, the lower oxygen contents in OLO-PVP-500 and OLO-PVP-600, compared to OLO-PVP-400, may be attributed to the increased evolution of gas phases including oxygen during the decomposition of PVP at a relatively high temperature.^31,32,50^ To confirm the chemical state and the ratio of Mn in the samples, the characteristic peaks of Mn2p were analyzed. The XPS Mn2p spectra of the samples could be completely fitted using the peaks related to Mn2p_1/2_ and Mn2p_3/2_ of Mn^4+^ and Mn^3+^ as shown in Fig. 7. In particular, the contents of Mn^3+^ in OLO-only, OLO-PVP-400, OLO-PVP-500, and OLO-PVP-600 were 15.3, 26.1, 25.5, and 25.7 at%, respectively. Compared to OLO-only, the samples prepared with PVP (OLO-PVP-400, OLO-PVP-500, and OLO-PVP-600) showed increased relative ratios of Mn^3+^. This demonstrates that the surface Mn^4+^ in Li_2_MnO_3_ can be chemically reduced to Mn^3+^ through the decomposition of PVP in the heating process under an N_2_ atmosphere. The chemically reduced Mn^3+^ surface state can lead to an enhanced electrical conductivity and Li^+^ ion motion, especially, expecting an improved rate capability in the charge–discharge process under a significantly high current density.^35,38,51–53^

**Table 1:** Contents of C, Mn, O, and Li in the samples measured using XPS analysis

at%	C	Mn	O	Li	Total
OLO-only	36.4	9.11	35.4	19.09	100.0
OLO-PVP-400	32.2	8.17	40.88	18.75	100.0
OLO-PVP-500	43.73	7.8	32.94	15.53	100.0
OLO-PVP-600	38.35	7.47	34.77	19.41	100.0

**Fig. 8:**
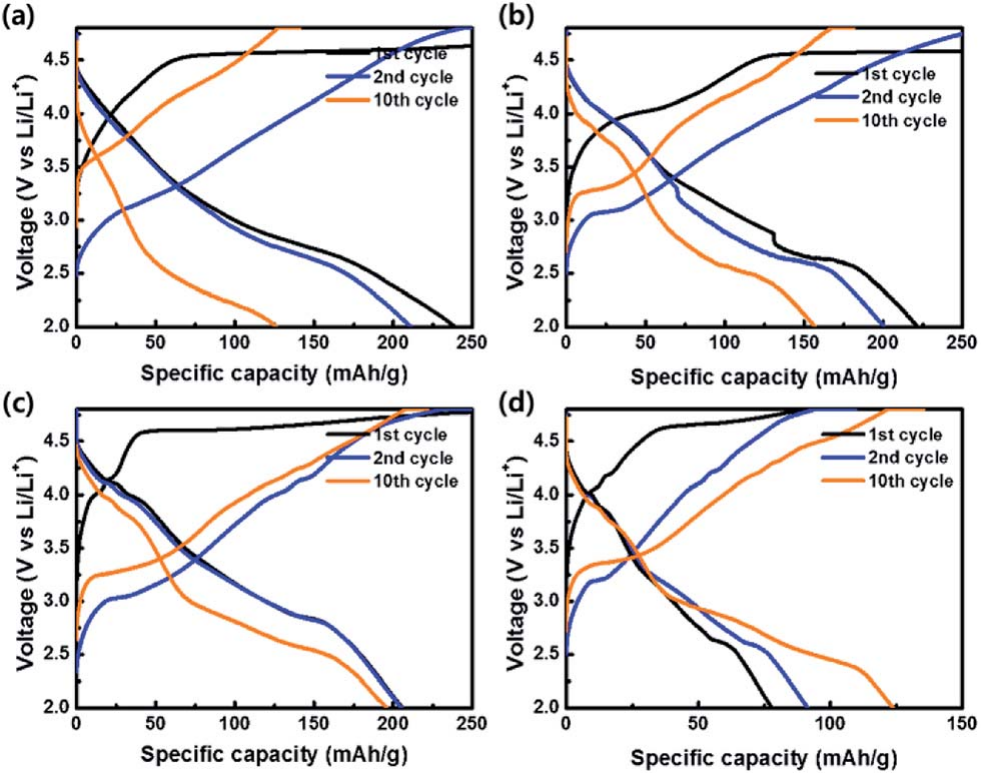
Charge–discharge curves of (a) OLO-only, (b) OLO-PVP-400, (c) OLO-PVP-500, and (d) OLO-PVP-600 measured at a current density of 20 mA g^−1^ in the potential range of 2.0–4.8 V *vs.* Li/Li^+^.

To evaluate the electrochemical properties of the coin-type cells with the samples as cathodes, charge–discharge curves were measured at a current density of 20 mA g^−1^ in the potential range of 2.0–4.8 V *vs.* Li/Li^+^, as shown in Fig. 8. All cells exhibited irreversible plateaus at 4.5 V *vs.* Li/Li^+^ during the 1st charge process due to an irreversible phase transformation caused by an oxygen loss. However, the irreversible phenomenon is essential for the activation of Li_2_MnO_3_ for the favorable transport of Li^+^ ion. After the activation process in the 1st cycle, no plateaus were shown in the curves and sloped curves appeared.^54–56^ The coulombic efficiencies of OLO-only, OLO-PVP-400, OLO-PVP-500, and OLO-PVP-600 during the 1st cycling were 59.3%, 38.1%, 61.9%, and 73.4%, respectively. On the other hand, the samples showed relatively sloped discharge curves in the 1st cycles due to a layered structure with Mn atoms between the transition metal layers. In addition, compared to OLO-only, OLO-PVP-400, OLO-PVP-500, and OLO-PVP-600 exhibited plateaus at ∼4.0 V in the discharge curves due to an oxygen deficiency caused by Mn^3+^, demonstrating the redox reaction between Mn^3+^ and Mn^4+^.^36^ The formation of Mn^3+^ can be attributed to the reduction of Mn^4+^ in Li_2_MnO_3_ during the heating process in the presence of PVP. All samples exhibited a voltage fading and a decreased capacity for 10 cycles, resulting from the side reaction between the electrode and electrolyte during cycling (Table 2).^7^ During the initial charge–discharge process at a relatively high voltage, gas evolution can occur due to the side reaction between the oxygen in the cathode and electrolyte. Furthermore, hydrogen fluoride (HF) generated by LiPF_6_ and H_2_O in the electrolyte at >4.5 V can attach to the electrode, resulting in voltage fading and the decreased capacity during the initial cycling.^57^ However, compared to OLO-only, the improved retention rates of OLO-PVP-400, OLO-PVP-500, and OLO-PVP-600 are attributed to the decreased polarization resulting from the reduced transformation in the OLO-PVP prepared in the presence of PVP.

**Table 2:** Discharge capacities of the samples as cathodes measured at a current density of 20 mA g^−1^ in the potential range of 2.0–4.8 V *vs.* Li/Li^+^

Capacity (mA h g^−1^)	OLO-only	OLO-PVP-400	OLO-PVP-500	OLO-PVP-600
1st cycle	239.3	221.4	206.0	77.9
2nd cycle	211.4	200.9	205.3	91.5
10th cycle	126.5	157.5	196.3	124.0

**Fig. 9:**
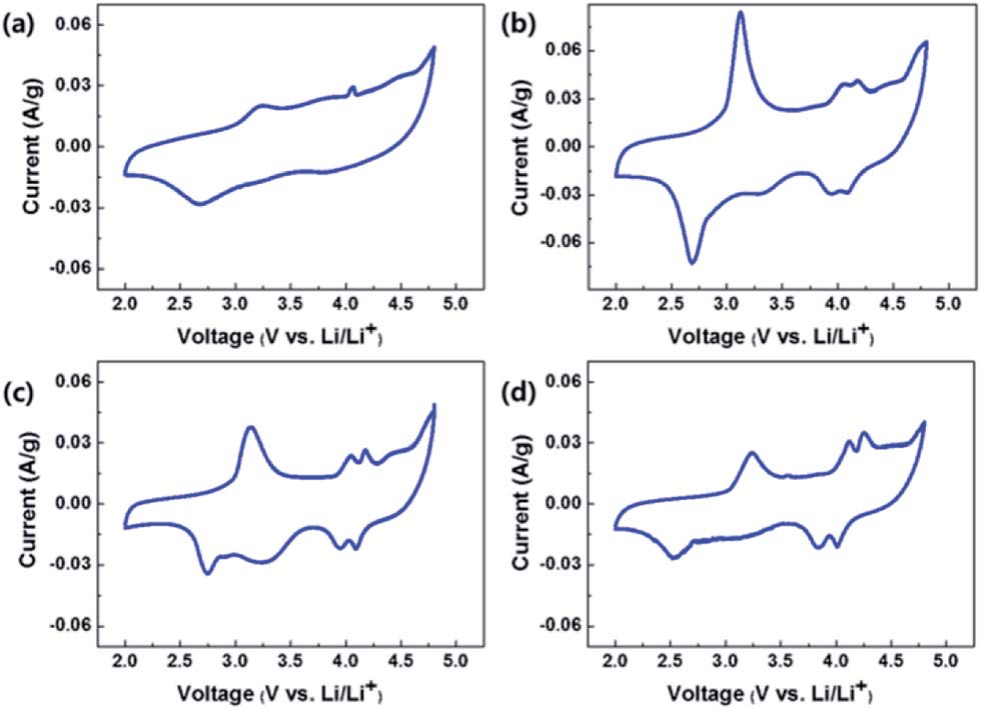
CVs of (a) OLO-only, (b) OLO-PVP-400, (c) OLO-PVP-500, and (d) OLO-PVP-600 measured at a scan rate of 0.1 mV s^−1^ in the potential range of 2.0–4.8 V *vs.* Li/Li^+^.

Fig. 9(a), (b), (c) and (d) show the CVs of OLO-only, OLO-PVP-400, OLO-PVP-500, and OLO-PVP-600, respectively, measured at a scan rate of 0.1 mV s^−1^ in the potential range of 2.0–4.8 V *vs.* Li/Li^+^. All samples exhibited oxidation peaks at ∼3.2 and ∼4.1 V during an oxidation scan and reduction peaks at ∼2.7 and ∼4.0 V during a reduction scan. The oxidation–reduction peaks in the CVs correspond to the voltages, which are expressed as plateaus in the charge–discharge curves in Fig. 8. In particular, compared to OLO-only and OLO-PVP-400, the OLO-PVP-500 and OLO-PVP-600 showed two sharp distinguishable peaks at ∼4.0 V, demonstrating improved diffusion rates related to the Li^+^ ionic and electronic motions due to the conductive carbon phases and the increased Mn^3+^ state.

**Fig. 10:**
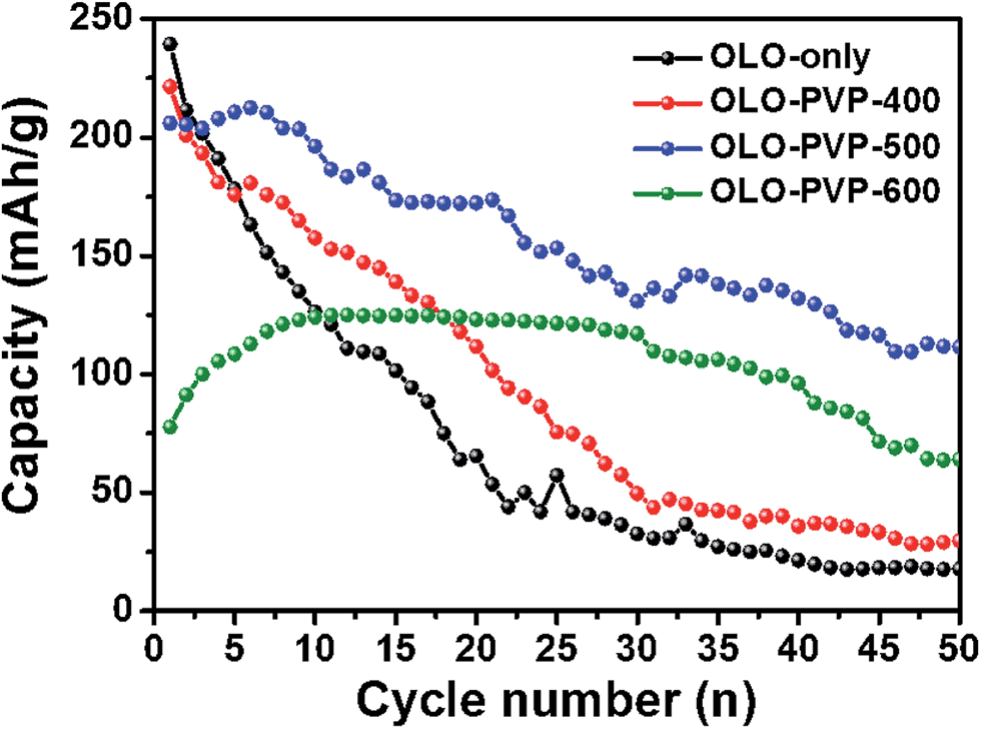
Cycling performance of the samples measured at a current density of 20 mA g^−1^ for 50 cycles.

Fig. 10 shows the cycling performances of the samples measured at a current density of 20 mA g^−1^ for 50 cycles. The discharge capacities of OLO-only, OLO-PVP-400, OLO-PVP-500, and OLO-PVP-600 for 20 cycles were 65.6, 111.6, 172.3, and 123.1 mA h g^−1^, respectively (Table 3). The retention rates of OLO-only, OLO-PVP-400, OLO-PVP-500, and OLO-PVP-600 for 20 cycles were 31.0%, 55.5%, 83.9%, and 134.5%, respectively. The discharge capacities of OLO-only, OLO-PVP-400, OLO-PVP-500, and OLO-PVP-600 for 50 cycles were 17.7, 29.7, 111.5, and 64.1 mA h g^−1^, respectively. The retention rates of OLO-only, OLO-PVP-400, OLO-PVP-500, and OLO-PVP-600 for 50 cycles were 8.4%, 14.8%, 54.3%, and 70.1%, respectively. Overall, OLO-PVP-500 and OLO-PVP-600 with relatively low specific surface areas exhibited superior cycling performance compared to OLO-only and OLO-PVP-400 with high specific surface areas. In general, the side reaction acting as the cause of the degradation of the cycling performance mainly occurs between the surface of an active material and an electrolyte.^7^ Thus, the relatively low surface areas of OLO-PVP-500 and OLO-PVP-600 can prevent the side reaction, showing an enhanced cycling performance. In addition, the increased relative ratios of Mn^3+^ in OLO-PVP-500 and OLO-PVP-600 can result in an improved Li^+^ ion motion, showing a superior charge–discharge performance. However, an excess of Mn^3+^ in the Mn-based cathode materials induces the Jahn–Teller effect (as the main cause of a deterioration in performance at the valence of <3.5), which can then deteriorate the stability of the Mn-based cathodes.^58,59^ Thus, in this study, the Jahn–Teller effect can be excluded. In particular, OLO-PVP-600 showed relatively low capacities of up to 10 cycles and gradually increased values from 10 cycles. This might be due to a slow activation process of the electrode heated with PVP in an N_2_ atmosphere at a relatively high temperature of 600 °C, followed by the heating in an O_2_ atmosphere at 600 °C. However, all samples showed a gradual capacity reduction during the process of 50 cycles due to a phase transformation in the cathode and a side reaction with an electrolyte.

**Table 3:** A comparison of discharge capacities and retention rates of the samples as cathodes measured at a current density of 20 mA g^−1^ for 20th and 50th cycles

Capacity (mA h g^−1^)	OLO-only	OLO-PVP-400	OLO-PVP-500	OLO-PVP-600
20th cycle	65.6	111.6	172.3	123.1
Retention ratio (%)	31.0	55.5	83.9	134.5
50th cycle	17.7	29.7	111.5	64.1
Retention ratio (%)	8.4	14.8	54.3	70.1

**Fig. 11:**
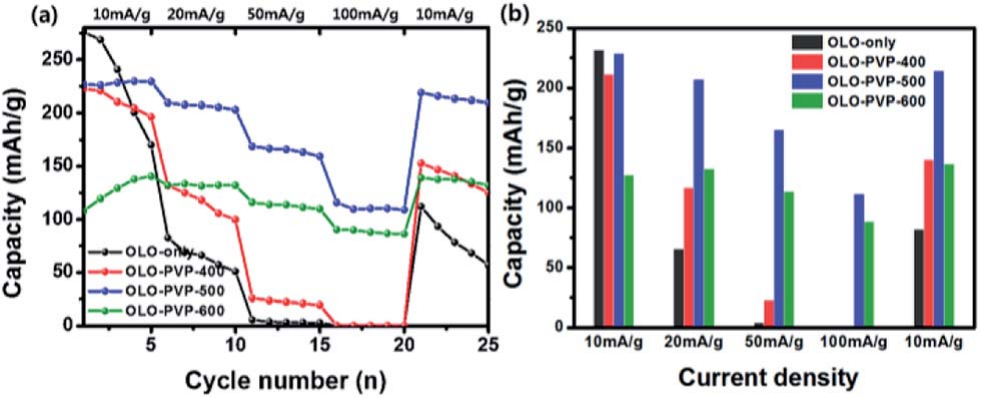
(a) Rate cycling performance for the samples measured at varying current densities from 10 to 100 mA g^−1^. (b) Average discharge capacities of the samples measured at 10, 20, 50, 100, and 10 mA g^−1^.

Fig. 11(a) shows a comparison of rate cycling performance for the samples measured at varying current densities from 10 to 100 mA g^−1^. As shown in Fig. 11(b), the average discharge capacities of OLO-only measured at 10, 20, 50, 100, and 10 mA g^−1^ were 231.3, 65.1, 3.9, 0.3, and 81.8 mA h g^−1^, respectively. The average discharge capacities of OLO-PVP-400 measured at 10, 20, 50, 100, and 10 mA g^−1^ were 211, 116.1, 22.5, 0.4, and 139.8 mA h g^−1^, respectively. The average discharge capacities of OLO-PVP-500 measured at 10, 20, 50, 100, and 10 mA g^−1^ were 228.1, 206.7, 164.8, 111.1, and 213.9 mA h g^−1^, respectively. The average discharge capacities of OLO-PVP-600 measured at 10, 20, 50, 100, and 10 mA g^−1^ were 127.1, 132.3, 113.1, 88.2, and 136.4 mA h g^−1^, respectively. In addition, as shown in Table 4, OLO-only and OLO-PVP-400 showed low retention rates with a serious reduction in capacity with increasing current density; no capacity was observed at >50 mA g^−1^. On the other hand, OLO-PVP-500 and OLO-PVP-600 maintained high retention rates with significantly high capacities with increasing current density, demonstrating an improved rate capability of OLO-PVP-500 and OLO-PVP-600. In particular, the superior rate performance of OLO-PVP-500 and OLO-PVP-600 can be attributed to a facilitated Li^+^ ion motion during the charge–discharge process, resulting from the existence of the conductive carbon phases on the electrode surface and the increased Mn^3+^ state caused by the decomposition of PVP. However, OLO-PVP-400 prepared in the presence of PVP exhibited an inferior rate performance due to the relatively high specific surface area and low content of the graphitic carbon structure. The electrochemical impedance spectroscopy (EIS) spectra of OLO-only and OLO-PVP-500 measured at 4.6 V after 2^nd^ and 20^th^ cycles were obtained (Fig. S2†). OLO-PVP-500 showed lower charge transfer resistance (*R*_ct_) values than OLO-only after 2^nd^ and 20^th^ cycles, demonstrating the improved Li^+^ ion motion during the cycling, because of the conductive carbon phases and the increased Mn^3+^ state.

**Table 4:** A comparison of average discharge capacities and retention rates of the samples as cathodes measured with varying current densities

	Capacity at 10 mA g^−1^	Capacity at 20 mA g^−1^	Capacity at 50 mA g^−1^	Capacity at 100 mA g^−1^	Capacity at 10 mA g^−1^
OLO-only	231.3 (100%)	65.1 (28.1%)	3.9 (6.0%)	0.3 (7.7%)	81.8 (35.4%)
OLO-PVP-400	211.0 (100%)	116.1 (55.0%)	22.5 (19.4%)	0.1 (1.8%)	139.8 (66.3%)
OLO-PVP-500	228.1 (100%)	206.7 (90.6%)	164.8 (79.7%)	111.1 (67.4%)	213.9 (93.8%)
OLO-PVP-600	127.1 (100%)	132.3 (104.1%)	113.1 (85.5%)	88.2 (78.0%)	136.4 (107.3%)

**Fig. 12:**
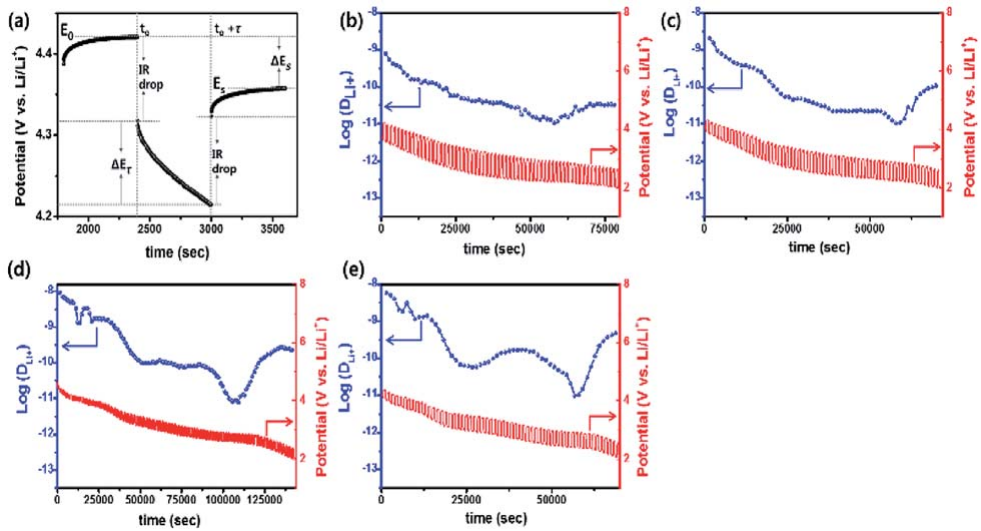
(a) Single titration profile of OLO-PVP-500 in the GITT during discharging. Plots of discharge potential and diffusion coefficient of (b) OLO-only, (c) OLO-PVP-400, (d) OLO-PVP-500, and (e) OLO-PVP-600 measured for three discharge cycles in the potential range of 2.0–4.8 V *vs.* Li/Li^+^.

GITT measurement was performed to evaluate the diffusion coefficients (*D*_Li^+^_) of Li^+^ ion in the cathodes (Fig. 12).^60^ The single titration profile of OLO-PVP-500 in the GITT measurement during discharging is shown in Fig. 12(a). The diffusion coefficient can be determined using the following equation:^61,62^
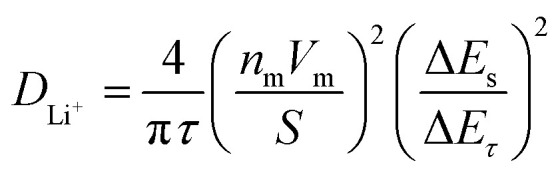
where *τ* is the current pulse time (s), *n*_m_ is mole (mol) number, *V*_m_ is a molar volume (cm^3^ mol^−1^), *S* is the contact area between the electrode and electrolyte (cm^2^), Δ*E*_s_ is the variation of steady-state voltage due to a current pulse, Δ*E*_*τ*_ is the difference between voltage and iR drop during a constant current pulse. The diffusion coefficients of OLO-only, OLO-PVP-400, OLO-PVP-500, and OLO-PVP-600 were measured for three discharge cycles in the potential range of 2.0–4.8 V (Fig. 12(b)–(e)). The average diffusion coefficients of OLO-only, OLO-PVP-400, OLO-PVP-500, and OLO-PVP-600 were 8.82 × 10^−11^, 1.96 × 10^−10^, 8.61 × 10^−10^, and 6.48 × 10^−10^ cm^2^ s^−1^, respectively. Compared to OLO-only and the typical cathodes of LiMn_2_O_4_ (10^−11^ to 10^−9^ cm^2^ s^−1^), LiCoO_2_ (10^−10^ to 10^−8^ cm^2^ s^−1^), and LiFePO_4_ (10^−14^ to 10^−15^ cm^2^ s^−1^), the OLO-PVP cathodes heated in the presence of PVP exhibited improved diffusion coefficients.^63^ In particular, the highest Li^+^ ion diffusion coefficient of OLO-PVP-500 and OLO-PVP-600 can be attributed to an enhanced electrical conductivity and Li^+^ ion motion caused by the existence of a graphitic carbon structure and increased relative ratios of Mn^3+^.

## 4. Conclusions

In summary, OLO cathode materials for high-performance LIBs were prepared by heating the as-prepared OLO in the presence of PVP acting as both a carbon source and reducing agent. The original crystal structure of Li_2_MnO_3_ was maintained during the heating process in the presence of PVP. However, the surface Mn^4+^ in Li_2_MnO_3_ could be chemically reduced to Mn^3+^ through the decomposition of PVP in the heating process under an N_2_ atmosphere. In particular, during the decomposition of PVP at 500 and 600 °C, the formation of graphitic carbon structure and the reduction of Mn^4+^ to Mn^3+^ were confirmed, resulting in an improved electronic conductivity and a facilitating Li^+^ motion. Thus, OLO-PVP-500 and OLO-PVP-600 showed a superior LIB performance with enhanced rate cyclability and high capacity retention.

## Conflicts of interest

There are no conflicts to declare.

## Supplementary Material

RA-009-C8RA10569C-s001
