# Supplementary material for: Role of polyvinylpyrrolidone in the electrochemical performance of Li2MnO3 cathode for lithium-ion batteries
Source: RSC Adv. 2019 Apr 2;9(18):10297–304. doi: 10.1039/c8ra10569c (PMC9062368; doi:10.1039/c8ra10569c)
Supplement: RA-009-C8RA10569C-s001 [file RA-009-C8RA10569C-s001.pdf]

Electronic Supplementary Material (ESI) for RSC Advances.
This journal is © The Royal Society of Chemistry 2019

Supporting Information

Role of polyvinylpyrrolidone in electrochemical performance of Li_2MnO_3 cathode for lithium-ion batteries

Ji-Eun Lee, Min-Cheol Kim, Sang-Hyun Moon, Eun-Soo Kim, Yeon-Kyung Shin, Sojeong Choi, Suk-Hui Kwon, Si-Jin Kim, Hye-Jin Kwon, and Kyung-Won Park*

Department of Chemical Engineering, Soongsil University, Seoul 156-743, Republic of Korea

* Corresponding author. Tel: 82-2-820-0613. Fax: 82-2-812-5378.

E-mail address: kwpark@ssu.ac.kr. (Prof. K.-W. Park)

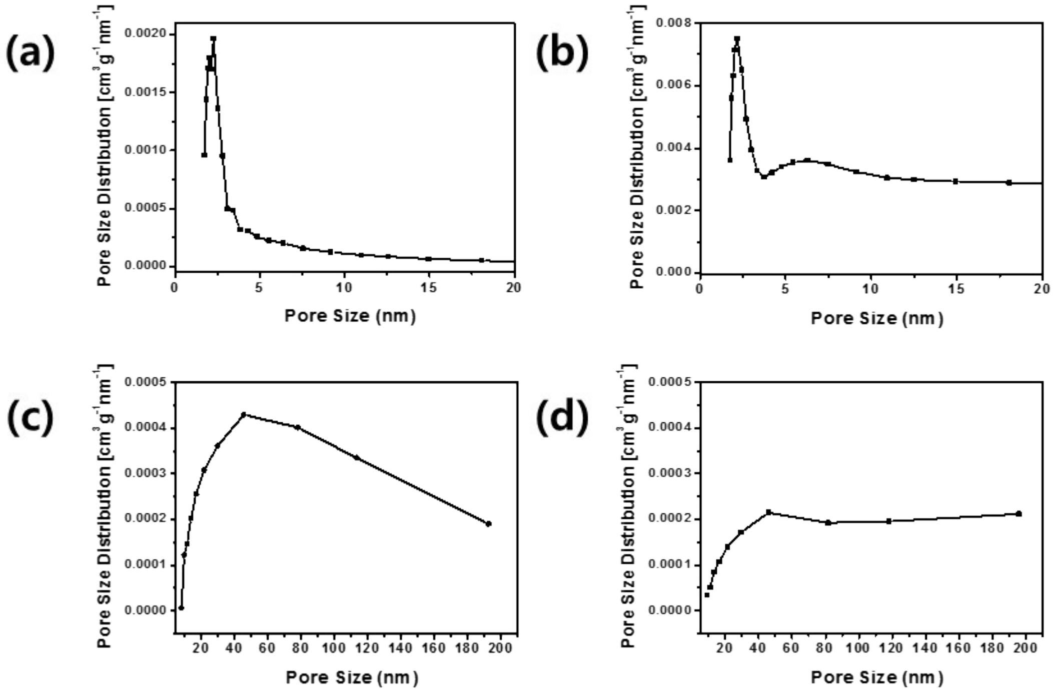

Figure S1. Pore size distribution curves of (a) OLO-only, (b) OLO-PVP-400, (c) OLO-PVP-500, and (d) OLO-PVP-600.

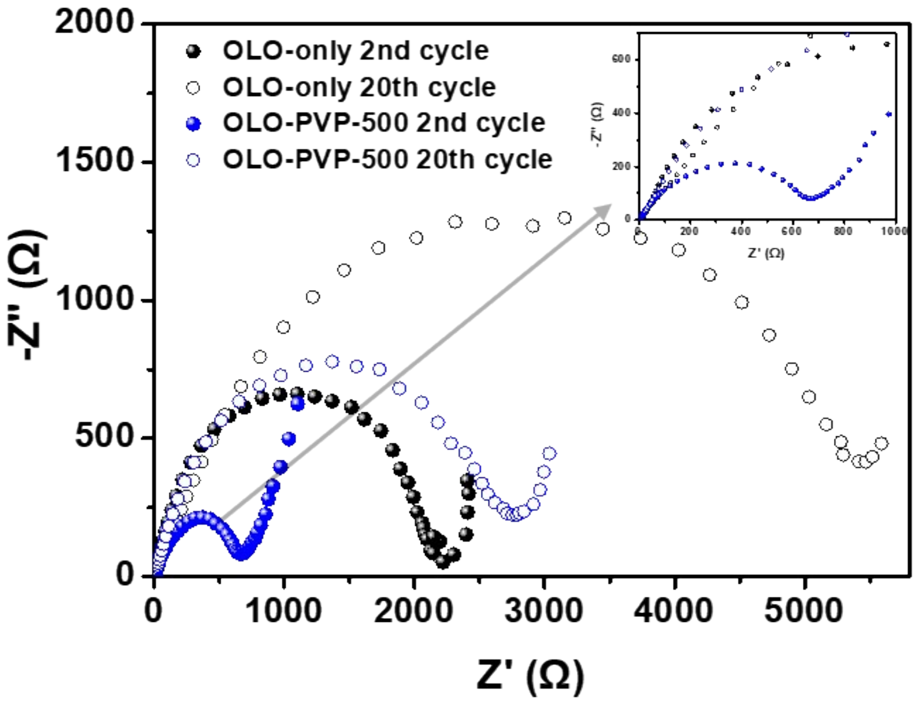

Figure S2. Electrochemical impedance spectroscopy spectra of OLO-only and OLO-PVP-500 measured at 4.6 V after 2nd and 20th cycles.
